# Generic characterization method for nano-gratings using deep-neural-network-assisted ellipsometry

**DOI:** 10.1515/nanoph-2023-0798

**Published:** 2024-01-16

**Authors:** Zijie Jiang, Zhuofei Gan, Chuwei Liang, Wen-Di Li

**Affiliations:** Department of Mechanical Engineering, The University of Hong Kong, Hong Kong, China

**Keywords:** spectroscopic ellipsometry, deep neural network, *in-situ* measurement, interference lithography, nanoimprint lithography, reactive-ion etching

## Abstract

As a non-destructive and rapid technique, optical scatterometry has gained widespread use in the measurement of film thickness and optical constants. The recent advances in deep learning have presented new and powerful approaches to the resolution of inverse scattering problems. However, the application of deep-neural-network-assisted optical scatterometry for nanostructures still faces significant challenges, including poor stability, limited functionalities, and high equipment requirements. In this paper, a novel characterization method is proposed, which employs deep-neural-network-assisted ellipsometry to address these challenges. The method processes ellipsometric angles, which are measured by basic ellipsometers, as functional signals. A comprehensive model is developed to profile nano-gratings fabricated by diverse techniques, by incorporating rounded corners, residual layers, and optical constants into an existing model. The stability of the model is enhanced by implementing several measures, including multiple sets of initial values and azimuth-resolved measurements. A simple compensation algorithm is also introduced to improve accuracy without compromising efficiency. Experimental results demonstrate that the proposed method can rapidly and accurately characterize nano-gratings fabricated by various methods, with relative errors of both geometric and optical parameters well controlled under 5 %. Thus, the method holds great promise to serve as an alternative to conventional characterization techniques for *in-situ* measurement.

## 1 Introduction

Optical scatterometry is a widely used, fast, and non-destructive characterization technique [1], [2] in the semiconductor industry for process monitoring to improve device yield [3]. Inverse scattering problems (ISPs), which extract nanostructure information from measured scattering signals [4], [5], are central to optical scatterometry. ISPs are typically treated as optimization problems, aiming to find a set of parameters including critical dimensions [6], [7] and optical constants [7], [8], [9], [10] that best match the theoretical optical responses with the measured ones. Several methods have been employed to solve ISPs, such as library search [11], non-linear regression [12], and heuristic optimization algorithms [13], [14]. However, these methods require numerical electromagnetic (EM) solvers, such as rigorous coupled wave analysis [15] (RCWA) method, finite-difference time-domain method [14], and finite element method [16], which are time- and resource-consuming. With the rapid development of the semiconductor industry, nanostructures in electronic devices are becoming increasingly complex [17], necessitating a highly efficient scatterometry technique for *in-situ* measurement.

Recent advancements in deep learning have opened up new possibilities for the rapid solution of inverse problems in nanophotonics [18]–[29]. Deep neural networks (DNNs) provide mathematical mappings from nanostructure parameters to optical responses, without the need for complex underlying physics. Consequently, DNNs can be thousands of times faster than numerical EM algorithms in predicting optical responses. Moreover, DNNs are composed of simple and differentiable operations, making it simple to analytically compute partial derivatives with respect to any input parameters. This allows for the application of gradient-based algorithms, resulting in improved performance in terms of both speed and accuracy. Several studies have reported the application of DNN-assisted optical scatterometry for nanostructure reconstructions using Muller-matrix ellipsometry [30] (MME), photonic dispersion [31] (PD), and other scattering signals [32], [33], [34]. However, these methods require expensive Muller-matrix ellipsometers or homebuilt measurement systems that are not commonly available. In contrast, standard ellipsometry (described by the Jones matrix formalism) is a more cost-effective and widely adopted scatterometry technique. Nevertheless, DNN-powered standard ellipsometry is currently limited to film thickness measurement [32], [34]. Furthermore, previously reported DNN-assisted optical scatterometry is only effective for specific processes [30], [31]. Several factors contribute to this issue. Firstly, the geometric models used for nanostructures are often oversimplified. For instance, nano-gratings are typically represented as isosceles trapezoids with sharp corners, which can adequately match grating profiles created by electron beam lithography (EBL) or reactive ion etching (RIE). However, this isosceles trapezoid model fails to fully capture the profiles of lithographic and nanoimprinted gratings, as it lacks important features such as rounded corners and residual layers. Secondly, previous works have predefined and fixed the refractive indices of the materials used in nanostructures, disregarding the fact that different fabrication processes typically involve different materials or categories of resists. Moreover, using predefined materials may lead to poor fitting quality for measured signals, as the actual refractive indices of the materials may deviate from the theoretical values during fabrication. Simultaneous fitting of both geometric and optical parameters provides more degrees of freedom for solving ISPs. While multiple processes are often employed in fabricating nanostructures, to the best of our knowledge, an optical scatterometry technique capable of characterizing nanostructures created by diverse fabrication methods has not been reported.

In this paper, we present a widely applicable characterization method for nano-gratings using standard ellipsometry assisted by DNNs. Compared to measurement equipment for MME and PD, standard ellipsometers have simpler configurations, making them more affordable and preferred by both laboratories and companies. Based on prior knowledge and sensitivity analysis, the profile of nano-gratings is modeled as an isosceles trapezoid with rounded corners and a residual layer, which better fits actual profiles and maintains high sensitivity. Additionally, the Cauchy dispersion formula is utilized to describe the materials of nano-gratings, providing more degrees of freedom to minimize fitting errors in solving ISPs. These additional grating parameters enable the proposed method to be effective for different fabrication processes. Furthermore, measures such as azimuth-resolved measurements and multiple initial values for optimization are adopted to alleviate the degeneration of injectivity and stability. Linear compensation is also employed to correct both systematic and random errors, which significantly improves accuracy. To demonstrate the effectiveness of the proposed method, nanoimprinted, etched, and lithographic nano-gratings were characterized. The relative errors of geometric parameters and refractive indices for all samples were found to be less than 5 %, indicating good performance.

## 2 Methodology

### 2.1 Measurement system

**Figure 1:**
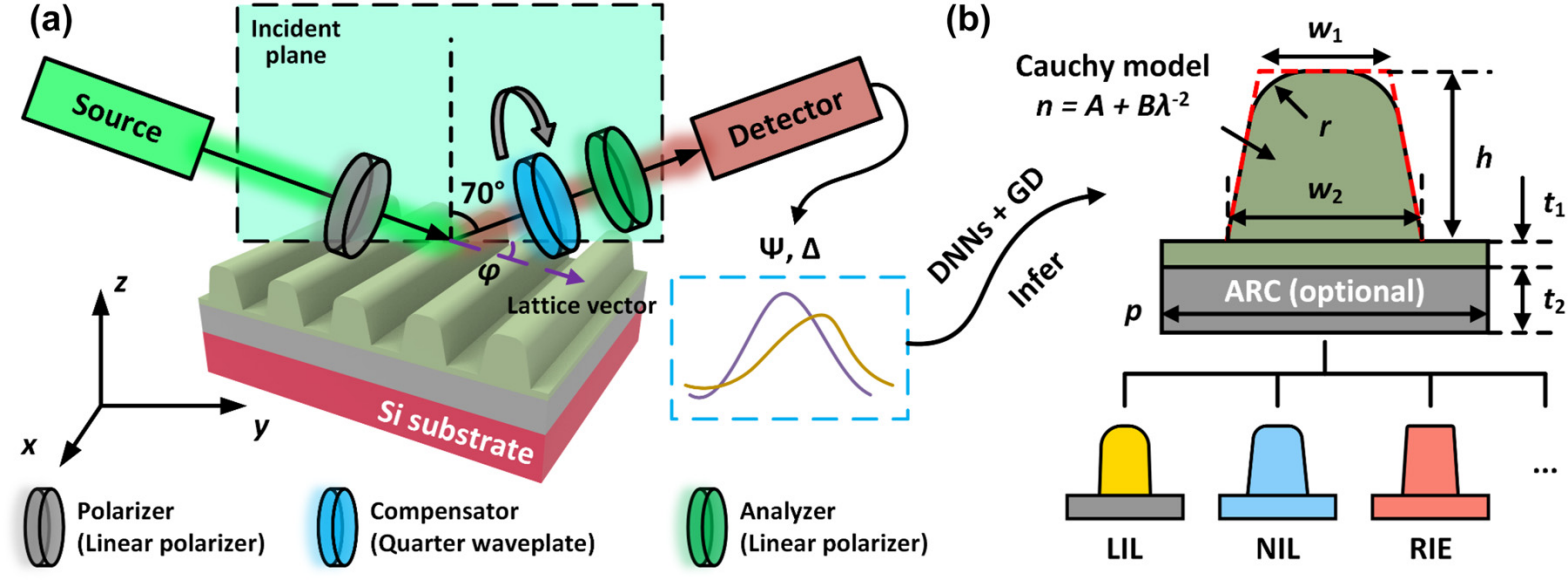
Overview of the DNN-assisted characterization method. (a) Schematics of the measurement system. (b) Applied model for nano-gratings with the incorporation of the rounded corners (*r*), residual layer (*t*_1_), and dispersion parameters (*A*, *B*).

Spectroscopic ellipsometry, using the Jones matrix formalism, relies on two ellipsometric angles: Ψ and Δ. These angles (Ψ and Δ) respectively represent the amplitude ratio and the phase difference between the *s*- and *p*-polarized reflected light. Figure 1(a) illustrates the configuration of the ellipsometer employed in this study for measurement. The light emitted from the source traverses the polarizer on the left arm and is converted into linearly polarized light, which is then incident on the sample with an angle of 70°. By rotating the compensator on the right arm, different polarization components of the reflected light are selectively filtered out for intensity measurement by the detector. Finally, the rotation angles of the compensator and the measured light intensities are utilized to derive the two ellipsometric angles. The available wavelength range for measurement spans from 400 nm to 900 nm. Additionally, the sample stage allows *z*-axis rotations enabling ellipsometry measurements to be carried out at various azimuthal angles (*φ*). In this context, the azimuthal angle is defined as the angle between the lattice vector of the nano-grating and the incident plane.

### 2.2 Model for nano-gratings

Nano-gratings created by different fabrication techniques exhibit unique features. As depicted in Figure 1(b), a model consisting of ten parameters is proposed to describe as many features as possible. Among these parameters, periodicity *p*, top line width *w*_1_, bottom line width *w*_2_, top rounded corner radius *r*, height *h*, residual layer thickness *t*_1_, and anti-reflection coating (ARC) thickness *t*_2_ are geometric parameters (*g*). Parameters *A* and *B* for the Cauchy dispersion formula determine the refractive index (*n*) of the material, and azimuthal angle *φ* is used to describe the orientation of nano-gratings during ellipsometry characterization. The incorporation of rounded corners in the model, in comparison with the commonly applied isosceles trapezoid model, enables better fitting of the actual profiles of lithographic nano-gratings (Section 5, Supplementary Materials). Notably, only top rounded corners are included in this model since ellipsometric data exhibits less sensitivity towards changes in the size of the bottom counterparts (Figure S1, Supplementary Materials). Additionally, a residual layer is placed beneath the nano-grating structure. The thickness of the residual layer is essential for optimizing some process parameters, such as the imprint resist thickness and etching time. The inclusion of parameters such as *r*, *t*_2_, *A*, and *B* enables our model to effectively describe nano-gratings fabricated using various techniques, including photolithography, EBL [31], nanoimprint lithography [35] (NIL), laser interference lithography [36], [37] (LIL), RIE [15], and others. The available ranges of grating parameters are listed in Table S1. It is noteworthy that the ranges of parameters *A* and *B* are large enough to cover a wide range of common materials, including photoresists, imprint resists, e-beam resists, and some optical materials (e.g., glass, fused silica, and alumina).

### 2.3 Forward mapping DNNs

**Figure 2:**
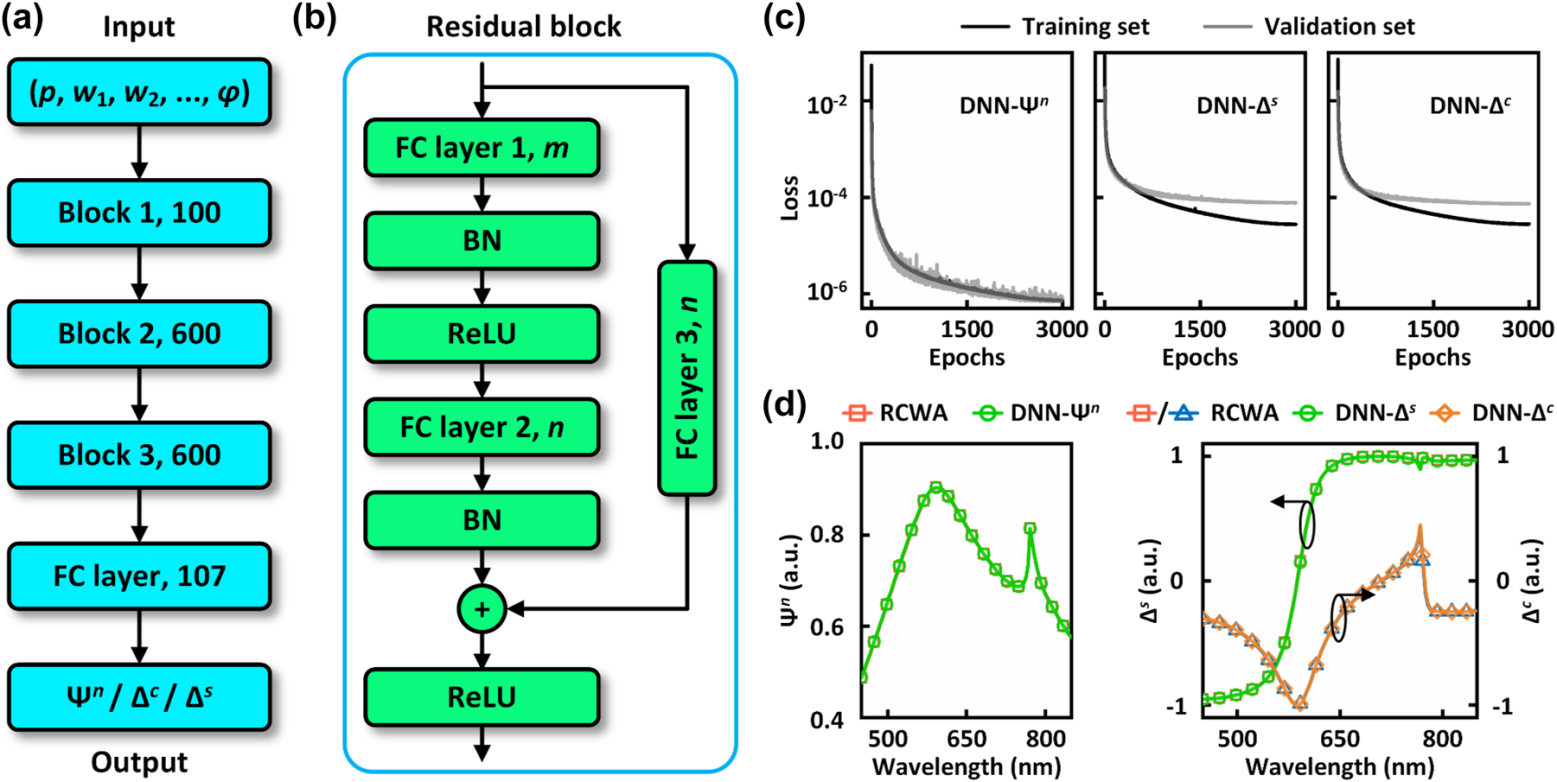
Architecture and training of deep neural networks (DNNs). (a) Architecture of DNNs. (b) Schematics of the residual block, where *m* and *n* indicate the number of neurons. FC layer 3 is optional when the size of the input is *n*. Block 1: *m* = *n* = 100. Block 2: *m* = 100, *n* = 600. Block 3: *m* = *n* = 600. FC, fully connected; BN, batch normalization; ReLU, rectified linear unit. (c) Training and validation losses for the DNNs. (d) Comparisons between the RCWA-simulated ellipsometric data and DNN-generated ellipsometric data of a nano-grating. Grating parameters are *p* = 405 nm, *w*_1_ = 154 nm, *w*_2_ = 203 nm, *r* = 48 nm, *h* = 179 nm, *t*_1_ = 33 nm, *t*_2_ = 0 nm, *A* = 1.475, *B* = 0.003 μm^−2^, *φ* = 12.3°.

For the more efficient solution of ISPs, numerical EM solvers are replaced by trained DNNs to establish forward mappings from grating parameters to theoretical ellipsometric data. As shown in Figure 2(a), the input to the DNNs consists of the ten aforementioned grating parameters. In order to mitigate the abrupt phase jump of ellipsometric angle Δ and reduce loss fluctuations during training, we utilize the sine (Δ^*s*^) and cosine (Δ^*c*^) values of Δ as the output labels. Simultaneously, Ψ is normalized (Ψ^*n*^ = Ψ/90°) to ensure that all ellipsometric parameters are within the same order of magnitude. The DNNs are constructed using the residual network [38] (ResNet) model, which facilitates convergence during training. Three DNNs, namely DNN-Ψ^*n*^, DNN-Δ^*s*^, and DNN-Δ^*c*^, are developed to predict different ellipsometric parameters. These DNNs share a common architecture comprising three residual blocks and a fully connected (FC) layer for adjusting the length of the output ellipsometric data. The configuration of the residual block is illustrated in Figure 2(b). Each residual block consists of two FC layers followed by batch normalization [39] (BN) layers and rectified linear unit [40] (ReLU) activation functions. Notably, the skip connection from the input to the output of the block provides additional gradients during the backward propagation, addressing the issue of vanishing gradients to some extent. The FC layer on the skip connection ensures compatibility between the shapes of the two added vectors.

To train the DNNs, a dataset comprising 660,000 sets of randomly generated grating parameters and their corresponding ellipsometric data simulated using our in-house RCWA code (Python) was created. The data collection lasted approximately 5 days using a supercomputing platform equipped with an Intel Xeon Gold 5218 central processing unit (16 cores) and 512 GB of memory for each node. The wavelength points (107 points) ranged from 450 nm to 850 nm, with an interval of 3.7 nm, consistent with the measurement points of the ellipsometer. Of the dataset, 80 % was used as the training dataset, while the remaining 20 % was used as the validation dataset. Due to limitations imposed by the GPU (Nvidia GeForce RTX 2060 SUPER), two sets of DNNs were trained separately for nano-gratings with and without an ARC layer (Section 3, Supplementary Materials). An Adam optimizer was used to train all DNNs with a batch size of 200. The learning rate began at 2 × 10^−3^ and gradually decayed to 1 × 10^−10^ after 3000 epochs, following the cosine annealing strategy. The training process for each DNN took approximately 10 h. Figure 2(c) shows the mean square error (MSE) losses of the training and validation datasets with respect to the training epochs when *t*_2_ = 0. Results for *t*_2_ > 0 can be found in Figure S2. The MSE losses for both training and validation datasets converged to a satisfactory level. Additionally, the generalization ability of the trained DNNs was tested by comparing the simulated and DNN-generated ellipsometric data for grating parameters that are not included in either the training or validation datasets. Figure 2(d) shows that the DNN-generated results align well with the simulated ones, indicating the high accuracy of prediction. Similar comparisons for gratings with an ARC layer can be found in Figure S2.

### 2.4 Generic characterization method for nano-gratings assisted by DNNs

Based on the trained DNNs and the GD algorithm, a novel and versatile characterization method for nano-gratings, known as DNN-assisted ellipsometry (DNNAE) method was developed. To ensure clarity, the grating parameters are categorized into three parts: geometric parameters (*g*) including *p*, *w*_1_, *w*_2_, *r*, *h*, *t*_1_, *t*_2_, optical parameters (*n*) consisting of *A* and *B*, and azimuthal angle (*φ*). It is worth noting that our method encompasses nearly twice as many parameters as previously reported methods. This expanded parameter space offers increased flexibility in solving ISPs. However, it also introduces challenges related to injectivity and stability, as the ellipsometric data measured under a single condition may correspond to multiple sets of grating parameters with slight variations. To address this issue, we adopt azimuth-resolved measurements (Section 6, Supplementary Materials). By acquiring ellipsometric data at various azimuthal angles for cross-validation, we can discern the unique solution that best aligns with all the ellipsometric data. To facilitate this process, azimuth-resolved measurements were performed using a fixed interval for the azimuthal angle (Δ*φ*). Consequently, only the starting measuring azimuth (*φ* ^0^) needs to be processed in the DNNAE method. The range for *φ* ^0^ is set from −30° to 10°, which offers convenience for alignment purposes. Mathematically, the proposed method aims to solve the following optimization problem:

(1) $$\begin{array}{ll} {\left(g,n,\varphi^{0}\right)}^{*} & =\mathrm{arg min}\frac{1}{N_{a}}\overset{N_{a}}{\underset{i=1}{\sum }}\|F\left(g,n,\varphi^{i}\right)-{\left(\Psi^{n,i},\Delta^{s,i},\Delta^{c,i}\right)\|}_{2} \end{array}$$

where the symbol * represents the optimal values, *N*_*a*_ represents the number of azimuthal angles, *F* denotes the forward mapping from grating parameters to ellipsometric data, which is achieved through the utilization of DNNs. Additionally, *φ* ^*i*^ = *φ* ^0^ + (*i* – 1)·Δ*φ* represents the *i*th measuring angle, and (Ψ^*n*,^ ^*i*^, Δ^*s*,^ ^*i*^, Δ^*c*,^ ^*i*^) corresponds to the measured ellipsometric data at *φ* ^*i*^.

**Figure 3:**
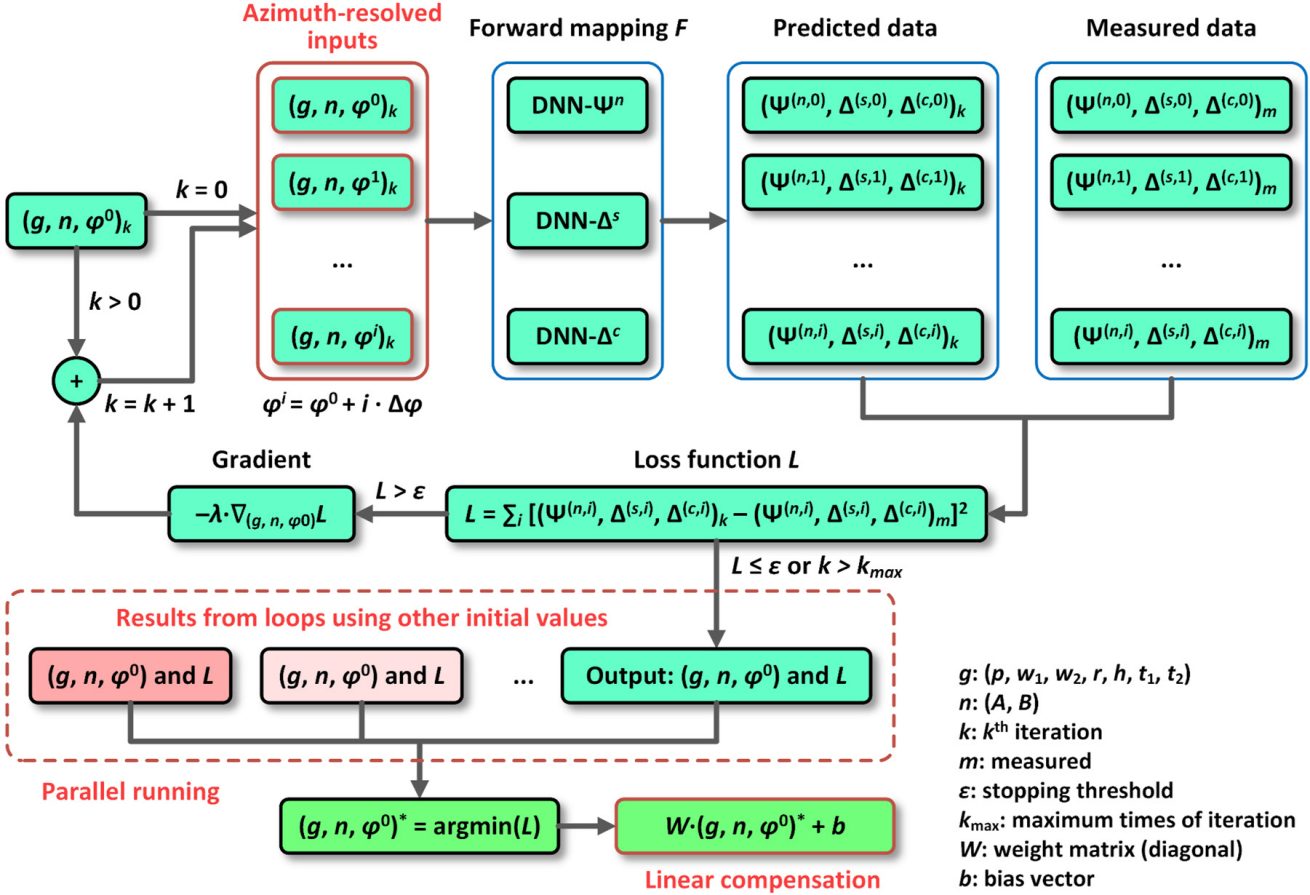
Architecture of the generic characterization method for gratings assisted by DNNs.

Figure 3 illustrates the architecture of the DNNAE method for solving Equation (1). Initially, a random set of grating parameters is generated within the predefined range. Then, this initial value is used as the candidate solution input into the DNNs, which produces the corresponding theoretical ellipsometric data. Here, the MSE loss is employed to measure the differences between the measured and theoretical ellipsometric data. The gradients of the loss are calculated through backward propagation to update the candidate solution. The step size of the update in each iteration is determined by the predefined rate *λ*. Subsequently, the algorithm proceeds to the next iteration, and this loop continues until the loss falls below the stopping threshold (*ε*) or the maximum number of iterations (*k*_max_) is reached. In practice, multiple sets of randomly generated initial grating parameters are optimized in parallel (dashed box in Figure 3) to mitigate the risk of local minima and enhance the stability of the DNNAE method (Section 6, Supplementary Materials). Furthermore, due to the parallel nature of this process, there is no significant increase in the time required. Once all parallel loops are completed, the optimal solution with the minimum MSE is selected. Lastly, a linear compensation algorithm is applied to the optimal solution to minimize the impact of systematic and random errors, such as the additional thickness of sputtered metal for SEM characterization and the dimensional shrinkage caused by the electron beam bombardment. The weight matrix and bias vector for linear compensation are determined using the least squares method and experimental data (Section 9, Supplementary Materials). With the assistance of widely applicable DNNs and measures to enhance both stability and accuracy, the DNNAE method shows promise as a versatile characterization method for nano-gratings fabricated through diverse processes.

## 3 Results and discussion

As a demonstration, three types of nano-gratings, namely nanoimprinted (thermal NIL), etched (RIE), and lithographic (LIL) nano-gratings were characterized. All nano-gratings were fabricated on silicon substrates. The nanoimprinted grating (sample 1) and etched grating (sample 2) both had residual layers, with the former made of imprint resist (poly butyl methacrylate, PBMA) and the latter composed of SiO_2_. The lithographic grating (sample 3), patterned using LIL, had no residual photoresist (AZ MIR 701), but an ARC (AZ BARLi-II 200) layer was precoated to minimize the reflection from the silicon substrate and ensure good morphology of nano-grating. Further details on the fabrication methods can be found in Figure S3.

Azimuth-resolved measurements were conducted at five different azimuthal angles (Section 7, Supplementary Materials) with an interval of 3°, for all samples. Prior to the iterations, one hundred sets of initial values were randomly generated. The initial update rate *λ* for the GD algorithm in the DNNAE method was set to 1, and it decreased by 0.1 every 50 iterations to promote better convergence. The stopping threshold was set to 1 × 10^−6^, and the number of iterations was limited to 2000. With the utilization of DNNs and parallel computing, the characterization of a grating sample takes approximately 58 s (using an Intel Core i7-10875H processor and 16 GB RAM), whereas conventional methods (RCWA and heuristic optimization algorithms) would require over 10 days to achieve similar performance. This time cost can be further reduced by employing GPU devices.

### 3.1 Characterization results of geometric parameters

**Figure 4:**
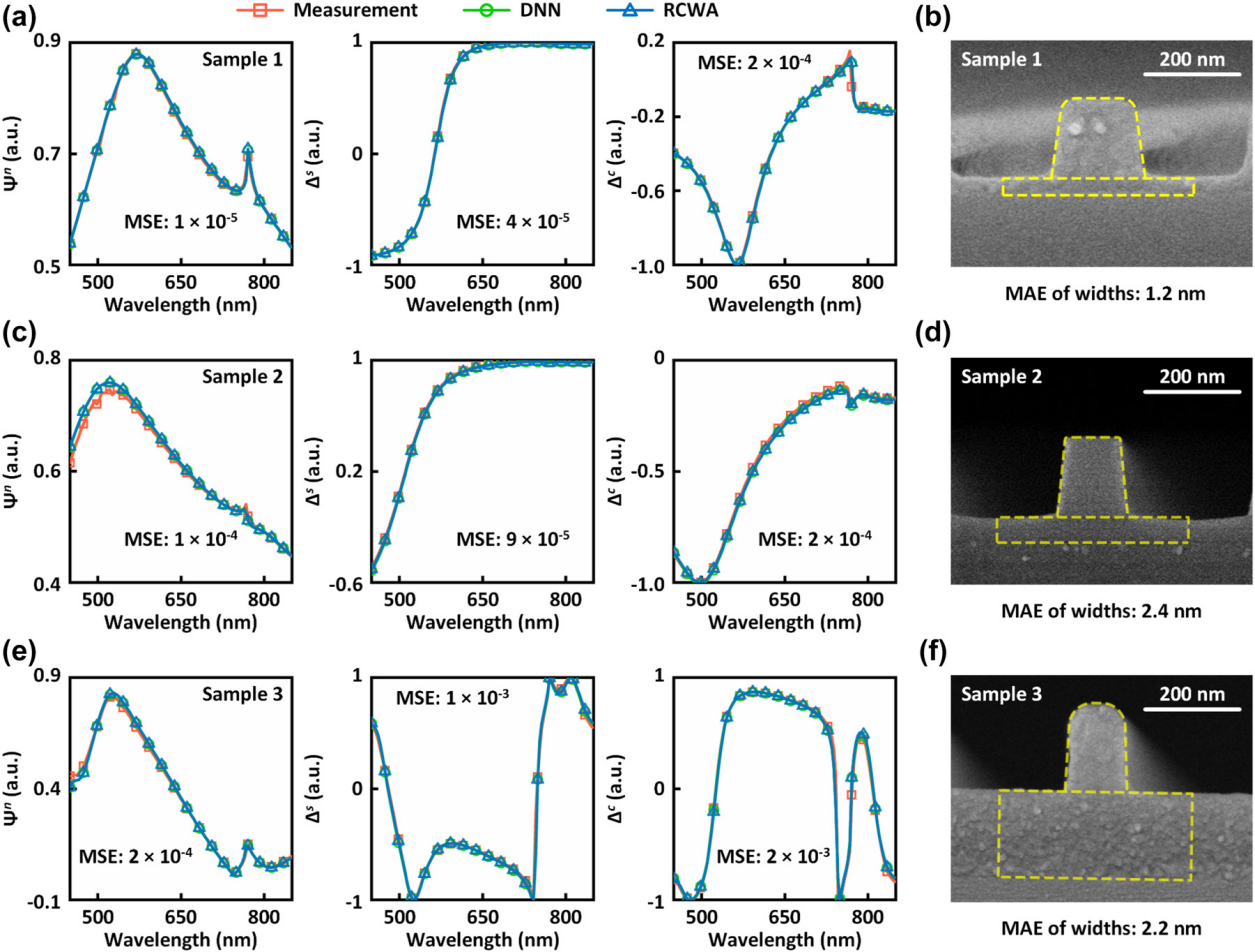
Characterization results of geometric parameters. (a), (c), (e) Comparison of ellipsometric data at the fifth measuring azimuthal angle for the nanoimprinted (sample 1), etched (sample 2), and lithographic (sample 3) nano-gratings, respectively. Measurement: ellipsometric data obtained from measurements. DNN: ellipsometric data generated by DNNs using the grating parameters inferred by the DNNAE method. RCWA: ellipsometric parameters simulated by RCWA using the grating parameters inferred by the DNNAE method. (b), (d), (f) Inferred profiles (yellow dashed lines) and SEM images for the nanoimprinted (sample 1), etched (sample 2), and lithographic (sample 3) nano-gratings, respectively.

Regarding the nanoimprinted grating (sample 1), Figure 4(a) shows the comparisons between the measured ellipsometric data and the DNN-generated ellipsometric data using the inferred grating parameters from the DNNAE method. Additionally, simulated ellipsometric data is also shown in blue for further verification. Notably, the ellipsometric data showcased in Figure 4 exclusively corresponds to the fifth measuring azimuth and displays the largest fitting MSEs when compared to all other measuring azimuths. The DNN-generated ellipsometric data is in excellent agreement with both simulated and measured ones, indicating the robust fitting capability of the DNNAE method. The MSEs between the measured and generated ellipsometric data are 1.3 × 10^−5^, 4.4 × 10^−5^, and 2.2 × 10^−4^, respectively for Ψ^*n*^, Δ^*s*^, and Δ^*c*^, which are comparable with the validation losses of DNNs. Table 1 lists the geometric parameters measured from SEM images and those inferred by the DNNAE method. After compensation, the relative errors are all less than 4 % and the largest absolute error is only 3 nm, indicating high accuracy. The characterization results of geometric parameters before compensation are listed in Table S2. Figure 4(b) presents a comparison of the SEM image and the inferred profile of the imprinted nano-grating. The inferred profile (yellow dashed lines) matches the SEM image perfectly, indicating that the DNNAE method accurately reconstructs the profile of the nano-grating. To quantitatively evaluate the difference between the inferred profile with the SEM-displayed one, we calculated the mean absolute error (MAE) of the grating widths (*δ*_*w*_) from the bottom to the top of the sample:

(2) $$\delta_{w}=\frac{1}{N_{w}}\overset{N_{w}}{\underset{i=1}{\sum }}\left|w_{i}^{*}-w_{i}\right|$$

where *N*_*w*_ = 100 reprsents the number of sampling points, while *w*_i_* and *w*_*i*_, respectively, indicate the widths of the inferred and SEM-displayed profile at the *i*th sampling point. The MAE of the grating widths for the imprinted nano-grating is only 1.2 nm. This outcome again highlights the high accuracy of the DNNAE method in reconstructing the geometry of nano-gratings.

**Table 1:** Measured (SEM) and inferred (DNNAE) geometric parameters for sample 1, and the corresponding relative errors.

Parameters	SEM (nm)	DNNAE (nm)	Relative error (%)
*p*	401	403	0.5
*w*_1_	152	151	0.7
*w*_2_	195	198	1.5
*r*	30	31	3.3
*h*	171	169	1.2
*t*_1_	35	36	2.9
*t*_2_	0	0	N/A

The characterization results for the etched nano-grating (sample 2) are depicted in Figure 4(c) and (d), showcasing a remarkable agreement between the DNN-generated, measured, and simulated ellipsometric data. The relative errors between measured and inferred geometric parameters are also well-controlled (Table 2). It is worth noting that the relatively larger error observed for the residual layer thickness (*t*_1_) can be attributed to the measurement uncertainty and the small thickness of the residual layer. The corresponding absolute error is only 3 nm. Additionally, the MAE of the grating widths for the etched sample is an impressive 2.4 nm.

**Table 2:** Measured (SEM) and inferred (DNNAE) geometric parameters for sample 2, and the corresponding relative errors.

Parameters	SEM (nm)	DNNAE (nm)	Relative error (%)
*p*	405	404	0.2
*w*_1_	119	116	0.3
*w*_2_	149	149	0
*r*	5	5	0
*h*	175	168	4.2
*t*_1_	50	53	6.0
*t*_2_	0	0	N/A

Moreover, we also conducted tests on lithographic samples. There is a noticeable difference between the measured ellipsometric data presented in Figure 4(e)–(c). However, the DNNAE method still effectively fits the measured data, resulting in minimal MSEs. The maximum relative errors of the geometric parameters are only 4.8 %, as indicated in Table 3. The accuracy of the DNNAE method remains unaffected by the presence of the ARC layer, demonstrating its robustness. Figure 4(f) showcases the profile inferred by the DNNAE method (indicated by yellow dashed lines), which aligns well with the nano-grating observed through SEM imaging. Furthermore, the MAE of the grating widths is only 2.2 nm, signifying the successful restore of the lithographic sample’s profile. These results indicate that despite the differences in features between the samples fabricated using different processes (e.g., the etched nano-grating having smaller rounded corners and a larger side-wall angle, while the lithographic sample exhibits the opposite), the DNNAE method can accurately infer the geometric dimensions from the measured ellipsometric data.

**Table 3:** Measured (SEM) and inferred (DNNAE) geometric parameters for sample 3, and the corresponding relative errors.

Parameters	SEM (nm)	DNNAE (nm)	Relative error (%)
*p*	407	407	0
*w*_1_	114	114	0
*w*_2_	125	131	4.8
*r*	48	50	4.2
*h*	186	187	0.5
*t*_1_	0	0	N/A
*t*_2_	184	183	0.5

Additionally, a stability test for the DNNAE method was also conducted. Thanks to the application of azimuth-resolved ellipsometric data and the incorporation of multiple initial solutions to solve ISPs, the characterization results of the geometric parameters exhibit commendable stability (Section 6, Supplementary Materials).

### 3.2 Characterization results for refractive index

**Figure 5:**
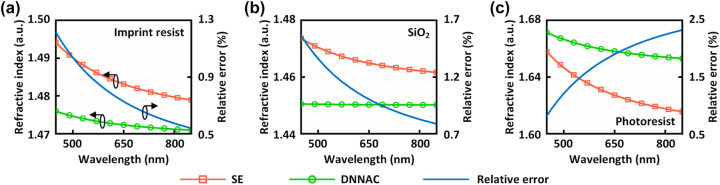
Characterization results of refractive indices. Comparisons of measured and inferred refractive indices for (a) imprint resist (PBMA), (b) SiO_2_, and (c) photoresist (AZ MIR 701). SE: refractive index determined from thin films using standard spectroscopic ellipsometry (SE). DNNAE: refractive index inferred by the DNNAE method. The relative errors of the refractive index are depicted by the solid lines.

In addition to the geometric parameters, the refractive index of nano-gratings can also be extracted from measured ellipsometric data. Figure 5(a)–(c) present a comparison between the refractive index inferred by the DNNAE method (indicated by circles) and the refractive index measured from thin films of corresponding materials (indicated by squares) using spectroscopic ellipsometry (SE). Slight deviations in the dispersion curves (<0.04) are observed. The corresponding relative errors are depicted using solid lines. The maximum relative errors of the refractive indices for imprint resist (PBMA), SiO_2_, and photoresist (AZ MIR 701) are 1.22 %, 1.57 %, and 2.28 %, respectively, indicating the high accuracy of the DNNAE method.

Additional characterization results for nanoimprinted, etched, and lithographic nano-gratings can be found in the Supplementary Materials (Section 10). These results exemplify the compatibility of the DNNAE method with various fabrication techniques, showcasing its ability to rapidly and accurately obtain the geometric and optical parameters of nano-gratings from measured ellipsometric data. Consequently, the DNNAE method holds promise as a robust tool for monitoring and optimizing various process parameters, such as exposure dose, resist thickness, etching time, and more.

## 4 Conclusions

In summary, we have developed a rapid, stable, accurate, cost-effective, and widely applicable DNN-assisted method for characterizing nano-grating profiles and refractive indices from measured ellipsometric signals. This method utilizes the ellipsometric angles obtained from inexpensive and commonly available equipment. We have enhanced the conventional model for nano-gratings by incorporating additional features such as rounded corners, residual layers, and refractive index. This modification enables the proposed method to characterize nano-gratings with diverse geometries and materials defined by different fabrication processes.

To validate the effectiveness of the DNNAE method, we conducted examinations using nanoimprinted, etched, and lithographic nano-gratings. Our experimental results demonstrated that the DNNAE method can achieve characterizations for nano-gratings within 1 min, with relative errors of less than 5 %. The stability of the characterization results was ensured through azimuth-resolved measurements and the utilization of multiple initial solutions in parallel optimizations.

By further refining the grating model and incorporating the Drude–Lorentz dispersion model, the DNNAE method can be easily extended to encompass additional manufacturing processes such as wet etching, material deposition, and others. The integration of DNNs and gradient descent methods has empowered us to effectively tackle highly complex ISPs. This work not only enriches the capabilities of ellipsometry for nanostructures but also facilitates the practical application of DNN-assisted ellipsometry in *in-situ* measurements and process monitoring.

## Supplementary Material

Supplementary Material Details
