# Supplementary material for: Generic characterization method for nano-gratings using deep-neural-network-assisted ellipsometry
Source: Nanophotonics. 2024 Jan 16;13(7):1181–9. doi: 10.1515/nanoph-2023-0798 (PMC11501600; doi:10.1515/nanoph-2023-0798)
Supplement: Supplementary file 1 — Supplementary Material Details [file j_nanoph-2023-0798_suppl_001.pdf]

Supplementary Materials

Generic Characterization Method for Nano-gratings Using Deep-Neural-Network-Assisted Ellipsometry

Zijie Jiang[1], Zhuofei Gan[1], Chuwei Liang[1], and Wen-Di Li[1]

[1]*Department of Mechanical Engineering, The University of Hong Kong, Hong Kong, China*

Correspondence author: Wen-Di Li (liwd@hku.hk)

1. Sensitivity Analysis

To optimize the model and measurement strategy, a sensitivity analysis was conducted using a method derived from the elementary effect method. This method applies a relative change on a parameter at a time while keeping all other parameters fixed. Subsequently, the output of the tested system before and after perturbation is compared to determine the effect (δ) caused by the change of the parameter:

$$\delta_i = \frac{1}{N_\lambda}\sum_{j=1}^{N_\lambda}\left\|F_j(x_1, x_2, \cdots, x_i + \Delta_i x_i, \cdots, x_n) - F_j(x_1, x_2, \cdots, x_i, \cdots, x_n)\right\|_2 \qquad \text{(S1)}$$

where F represents the forward mappings created by deep neural networks (DNNs) for ellipsometric parameters Ψ^n, Δ^s, and Δ^c, x corresponds to the grating parameters, and Δ_i is the relative change (5%) applied on the ith parameter. Additionally, $N_\lambda = 107$ is the number of wavelength points, and j denotes the jth wavelength point. It is worth mentioning that the mean square error (MSE) is utilized to evaluate the effect of the perturbation, as it is also employed in the training and optimization processes, facilitating convenient comparisons. Similarly, the relative change is used because the main text mostly utilizes relative error for accuracy evaluation. In practice, multiple different starting points for grating parameters are randomly generated to include as many samples as possible, which is advantageous for a comprehensive and precise evaluation of sensitivity. The sensitivity measure is determined by the mean value of δ_i:

$$\mu_i = \frac{1}{N_p}\sum_{k=1}^{N_p}\delta_i^k \qquad \text{(S2)}$$

where N_p represents the number of the starting points for grating parameters. The sensitivity

measure (μ_i) represents the variation in the ellipsometric data resulting from a Δ_i relative change on the ith grating parameter. Figure S1 depicts the simulated sensitivity measures with respect to azimuths for a subset of grating parameters, namely top width (w_1), bottom width (w_2), top corner radius (r_1), bottom corner radius (r_2), height (h), and residual layer thickness (t_1). The variations in Δ^s and Δ^c are found to be significantly larger than that of Ψ^n, indicating the greater importance of Δ^s and Δ^c in the characterization process. The sensitivity for linewidth-related parameters (w_1, w_2, r_1, r_2) exhibits similar behaviors, with peak values occurring at approximately 30° azimuth for both Δ^s and Δ^c. Additionally, the sensitivity of thickness-related parameters (h, t_1) is approximately one order of magnitude larger than that of linewidth-related parameters. Owing to the symmetry of gratings, the sensitivity for negative azimuthal angles is identical to that for positive angles. Consequently, a measurement range of -35° to 35° for the azimuthal angle was chosen to strike a balance between sensitivity and the complexity of training the DNNs. Within this range, the minimum sensitivity for most grating parameters (except r_2) exceeds the validation losses of the trained DNNs (9×10^{-7} for DNN-Ψ^n, 1×10^{-4} for DNN-Δ^s, and 9×10^{-5} for DNN-Δ^c), indicating the accuracy of the DNNs in detecting 5% changes. However, due to the low sensitivity, the rounded corners at the bottom were neglected in the final model (Figure 1) utilized for the deep-neural-network-assisted ellipsometry (DNNAE) method to ensure the accuracy of the bottom width measurement.

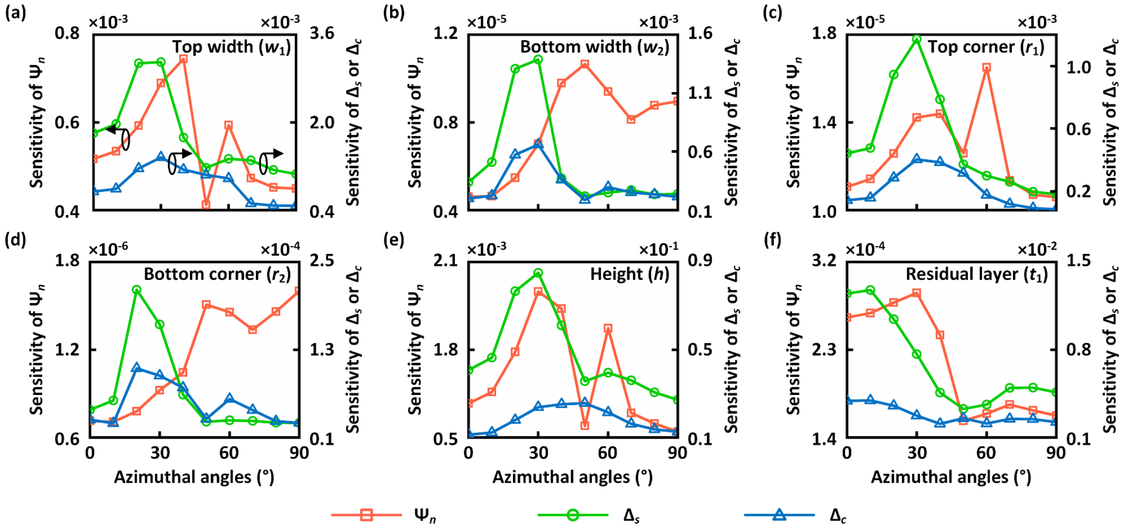

Figure S1. Sensitivity analysis for geometric parameters. (a) Top width (w_1). (b) Bottom width (w_2). (c) Top rounded corner radius (r_1). (d) Bottom rounded corner radius (r_2). (e) Height (h). (f) Residual layer thickness (t_1).

2. Ranges of Grating Parameters

Ranges of grating parameters are provided in Table S1. In practice, the ratio of r to w_1 (r / w_1) is employed to regulate the size of top rounded corners, and it is crucial to ensure that this ratio remains smaller than 0.5 to maintain the continuity of grating profiles.

Table S1. Ranges of grating parameters. Low.: lower limit. Upp.: Upper limit. The units of p, w_1, w_2, r, h, t_1, and t_2 are nm. The units of B and φ are μm^{-2} and °, respectively.

	p	w_1	w_2	r	h	t_1	t_2	A	B	φ
**Low.**	400	80	80	5	150	0	180	1.45	1×10^{-4}	-35
**Upp.**	410	205	305	150	200	50	200	1.65	2×10^{-2}	35

3. Training of Deep Neural Networks (DNNs)

The construction and training of DNNs were conducted using PyTorch on a computer work-station equipped with an AMD Ryzen 3970X central processing unit (CPU), an Nvidia GeForce RTX 2060 SUPER graphic processing unit (GPU), and 64 GB of RAM. Due to the limitations of the GPU's compute capability, the dataset was divided into two parts based on the thickness (t_2) of the anti-reflection coating (ARC), with 300,000 data points for $t_2 = 0$ and 360,000 data points for $t_2 > 0$. These subsets were separately fed into the DNNs for training. Figures S2(a) – (c) illustrate the training and validation losses for gratings with an ARC layer ($t_2 > 0$). All losses converged well to values lower than the minimum sensitivity shown in Figure S1. As depicted in Figures S2(d) – (f), the trained DNNs can precisely predict the ellipsometric data for gratings with an ARC layer.

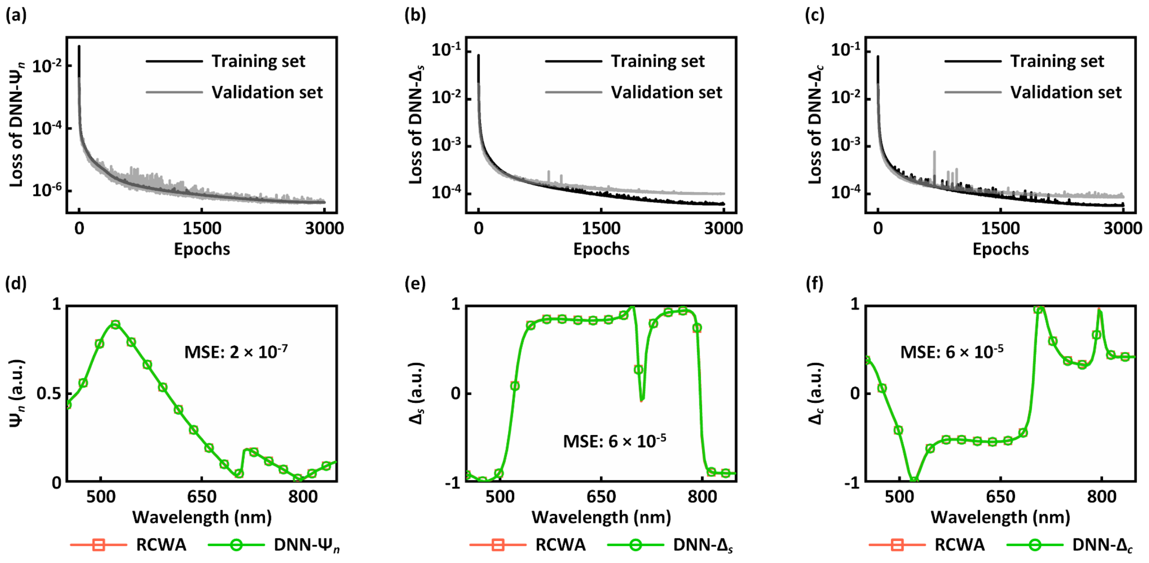

Figure S2: Training of deep neural networks (DNNs) (a) – (c) Training and validation losses of DNNs for nano-gratings with an ARC layer. (d) – (f) Comparisons between the RCWA-simulated and DNN-generated ellipsometric data of a nano-grating. Grating parameters are p = 403 nm, w_1 = 117 nm, w_2 = 131 nm, r = 50 nm, h = 191 nm, t_1 = 0 nm, t_2 = 189 nm, A = 1.601, B = 0.012 μm^{-2}, φ = 25.1°.

4. Fabrication Methods

Figure S3(a) illustrates the procedures for fabricating nano-gratings using laser interference lithography (LIL). Initially, the ARC (AZ BARLi-II 200) and photoresist (AZ MIR 701, diluted with 1:1 using propylene glycol methyl ether acetate) were spin coated onto the silicon substrate in a sequential manner. The photoresist was then exposed using a homebuilt LIL system, with different exposure doses utilized to create various grating profiles. Lastly, the lithographic samples were obtained after post-baking (110°C, 60s) and development (AZ 726, 60s).

As shown in Figure S3(b), etched nano-gratings were fabricated using a silicon oxide wafer produced by thermal oxidation as the substrate. Firstly, LIL was performed to define the pattern on the photoresist, following the same procedures as shown in Figure S3(a). The ARC was then etched using the photoresist mask. To enhance selectivity, a thin layer of chromium was added to the photoresist gratings through shadow evaporations with evaporation angles of +60° and -60°. Subsequently, the desired depth of SiO_2 was etched using CF_4 and O_2 in the reactive ion etching (RIE) process. Finally, the chromium layer and polymers (photoresists and ARC) were sequentially removed using chromium etchant and RCA-1 clean.

Figure S3(c) presents the fabrication method for nanoimprinted nano-gratings. Imprint molds were prepared using a commercial UV-curing polymer, namely OrmoStamp, for improved performance. The master mold was a SiO_2 grating fabricated through the method shown in Figure S3(b). After the anti-sticking treatment, uncured OrmoStamp was dispensed to the master. A glass substrate, treated with an adhesion promoter (OrmoPrime 08), was then placed onto the liquid OrmoStamp and master mold. The force of gravity facilitated the spreading and filling

of the liquid OrmoStamp within the trenches of the nano-gratings. The liquid OrmoStamp was subsequently cured using a UV lamp and separated from the master mold. After a hard baking process (110°C, 30 min), a reusable polymer imprint mold was obtained. Next, the homemade imprint resist (0.8 g poly butyl methacrylate dissolved in 19.2 g propylene glycol methyl ether acetate) was spin coated onto the silicon substrate, followed by thermal nanoimprint lithography (NIL) conducted at a temperature of 100°C. The applied pressure was 5.0 MPa, and the process lasted for 10 minutes. After cooling, the nano-grating was separated from the mold.

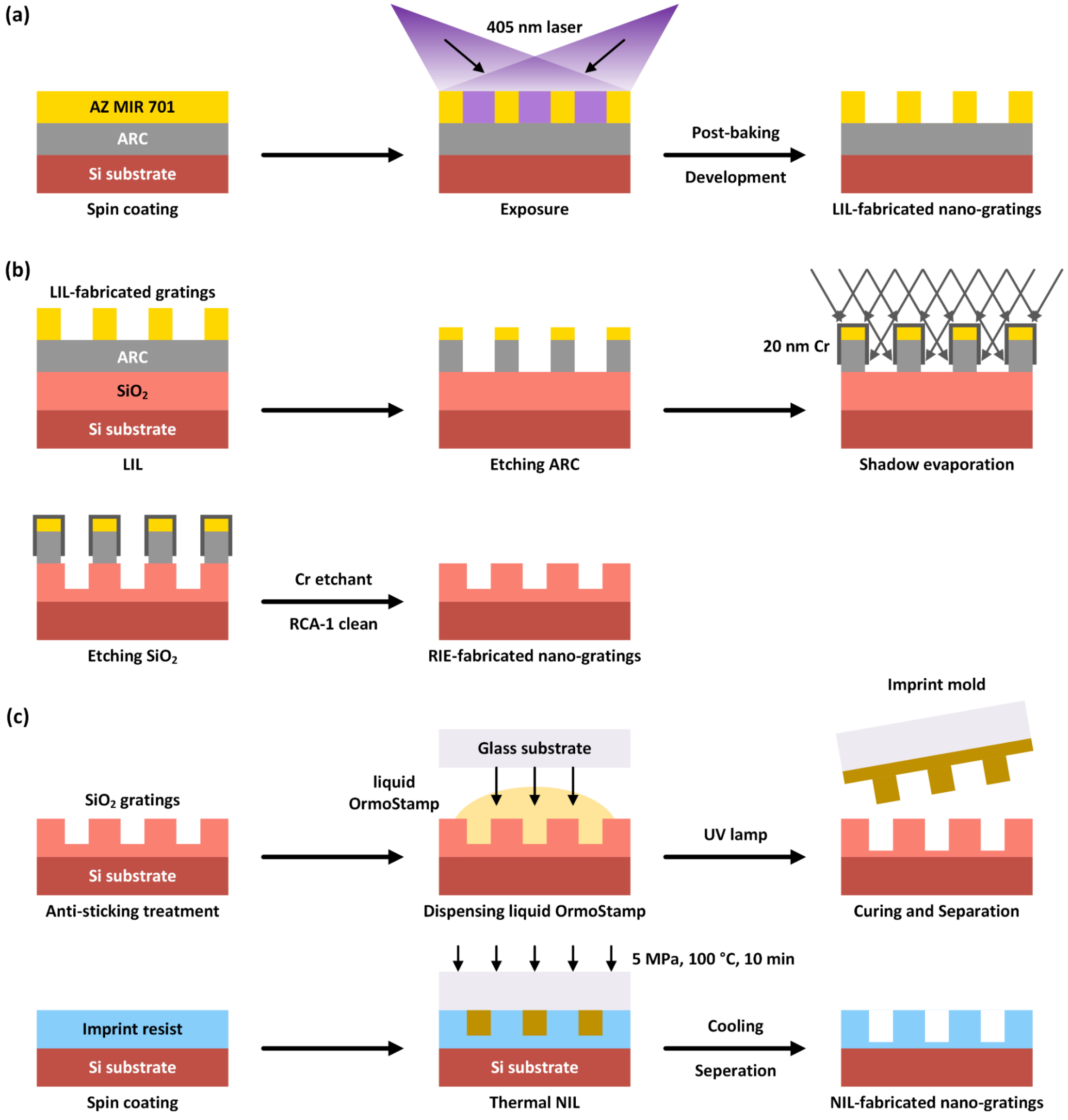

Figure S3: Fabrication methods for tested samples.

5. Comparisons of Models

The proposed model in the main text is compared to the isosceles trapezoid model to demonstrate the significance of top rounded corners. Simulated ellipsometric parameters obtained through rigorous coupled-wave analysis (RCWA) are used as the fitting target to minimize the influence of external factors such as noise and systematic errors in measurement. In order to replicate the isosceles trapezoid model, we set the upper limit of the rounded corner radius (r) to 0 before implementing the DNNAE method. Figures S4(c) – (d) reveal that although the ellipsometric data is restored, the inferred profile using the isosceles trapezoid model fails to perfectly match the actual profile (gray area), particularly near the top rounded corners. Consequently, the mean absolute error (MAE) of the grating widths (12.2 nm) is significantly larger compared to the MAE (1.5 nm) of the grating widths inferred by the DNNAE method using the modified model.

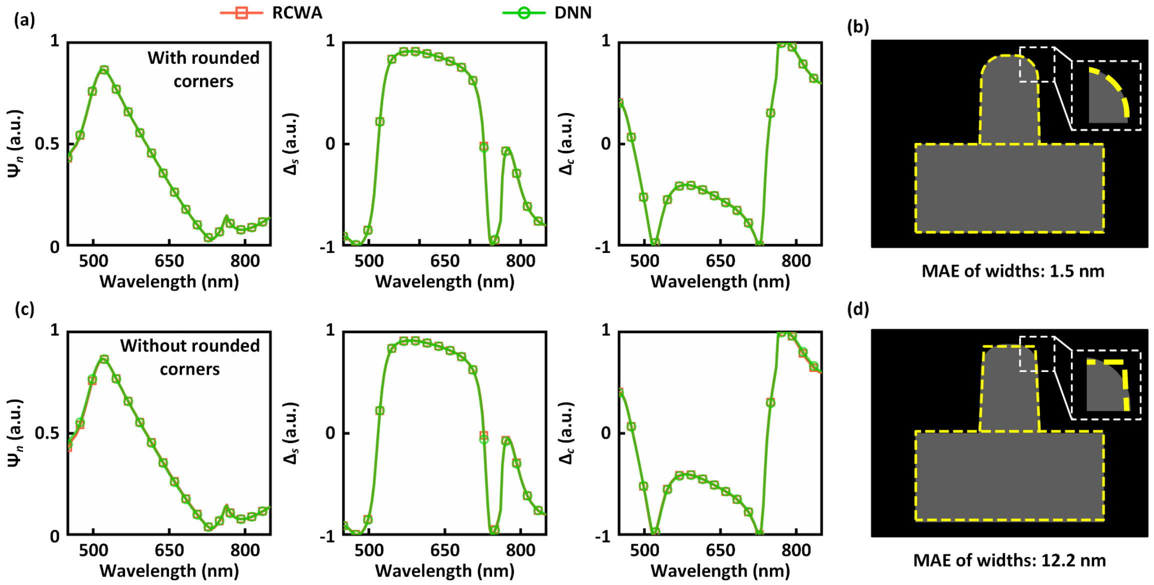

Figure S4: Characterization results using different models for nano-gratings. (a) – (b) Model proposed in the main text. (c) – (d) Isosceles trapezoid model.

6. Stability of the DNNAE method

To assess stability, we ran the DNNAE method 100 times for sample 1 from the main text. Figure S5(a) displays the deviations (subtracted by the average values) of the obtained geometric parameters from each run, providing a clear visualization of the fluctuations. Remarkably, the deviations of the obtained parameters are minimal and exhibit an inverse proportionality to the sensitivity. The corresponding standard deviations (σ) for all parameters are below 0.2 nm, indicating a high level of stability.

To showcase the advantages of azimuth-resolved ellipsometry, we conducted the same stability test using ellipsometric data measured under a single azimuthal angle (φ^0). As depicted in Figure S5(b), the oscillation zone of the results becomes much wider, and the standard deviations are several times larger compared to those listed in Figure S5(a). This observation emphasizes the potential for improved stability in our DNNAE method through the application of azimuth-resolved ellipsometry.

Furthermore, we investigated the influence of initial values. Figure S5(c) illustrates the deviations of geometric parameters when only one set of initial solutions was used for optimization. As anticipated, the standard deviations are one order of magnitude larger than those listed in Figure S5(a) due to the presence of local minimum points. Different starting points yield distinct optimization trajectories. The use of different initial values in parallel optimization increases the likelihood of converging to the global optimum, consequently enhancing the stability.

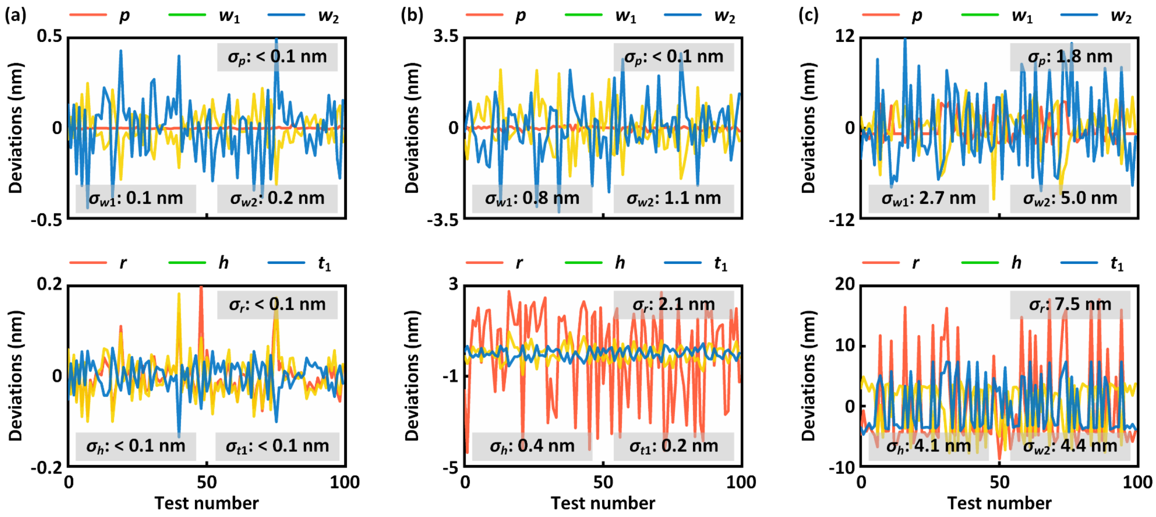

Figure S5: Stability assessment of the DNNAE method under various conditions. (a) Deviations of obtained geometric parameters using ellipsometric data from five azimuthal angles and a hundred sets of initial values. (b) Deviations of obtained geometric parameters using ellipsometric data from only one azimuthal angle and a hundred sets of initial values. (c) Deviations of obtained geometric parameters using ellipsometric data from five azimuthal angles and only one set of initial values.

7. Selecting the number of azimuthal angles

The stability of the DNNAE method is significantly influenced by the number of azimuthal angles and initial values, as discussed in Section 6 of the Supplementary Materials. Choosing the appropriate number of azimuthal angles and initial values is crucial in balancing stability and time consumption. The number of initial values is only related to the time required to solve the inverse scattering problems (ISPs), and its impact is minimal due to the parallel nature of the DNNAE method. However, increasing the number of azimuthal angles results in additional time consumption for both measurement and ISP solution. Therefore, it is essential to minimize the number of azimuthal angles while ensuring sufficient stability.

Since the standard deviations of obtained geometric parameters should be at least one order of magnitude smaller than the accuracy (several nanometers), as shown in Figure S5(a), we utilized ellipsometric data measured under five azimuthal angles in this study. The time consumption was around 1 minute on our computing platform (Intel Core 10875H central processing unit, 16 GB of memory), which was deemed acceptable. It is important to note that measuring ellipsometric data under five different azimuthal angles using a manual rotation stage took about 10 minutes. The measurement time could be significantly reduced by implementing a motorized stage.

In fact, the minimal number of azimuthal angles required for stability primarily depends on the number of grating parameters. When dealing with a larger number of grating parameters, it is generally necessary to measure ellipsometric data under more azimuthal angles due to the expansion of the parameter space and increased complexity. Conversely, when considering fewer

grating parameters, the need for measuring ellipsometric data under multiple azimuthal angles is reduced. To demonstrate this relationship, we conducted stability tests by keeping the top width (w_1) and bottom width (w_2) constant. Figure S6 shows that by using ellipsometric data measured under just three azimuthal angles, adequate stability can be achieved when considering only eight grating parameters. Therefore, in practical applications where fewer grating parameters are required, the number of azimuthal angles can be decreased accordingly.

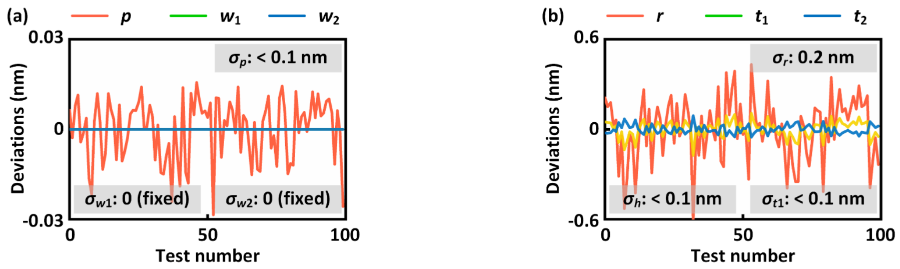

Figure S6: Stability assessment of the DNNAE method with consideration of fewer grating parameters. Ellipsometric data from three azimuthal angles and a hundred sets of initial values were utilized (a) Deviations of period p, top width w_1 (fixed at a constant value), and bottom width w_2 (fixed at a constant value). (b) Deviations of top corner radius r, height h, and residual layer thickness t_1.

8. Characterization Results Before Compensation and Error Analysis

Table S2 presents a comparison between the inferred (before compensation) and measured geometric parameters. The observed differences can be attributed to several factors. The primary systematic error arises from the additional thickness (up to 10 – 20 nm) of the sputtered metallic layer utilized for conductivity enhancement in SEM. Simultaneously, the major random error stems from the shrinkage of dimensions (approximately 0 – 20 nm) caused by the bombardment of the electron beam during imaging. Furthermore, the manual measurement of dimensions from SEM images also introduces deviations of several nanometers. Other factors, including sample tilting in SEM characterization, deformations caused by cracks, and the convergence angle of the light source, have tiny influences on the overall errors.

Tabel S2. Characterization results of geometric parameters before compensation. SEM: geometric parameters (nm) measured from SEM images. DNNAE: uncompensated geometric parameters (nm) inferred using the DNNAE method. RE: relative error (%).

**Params.**	**Sample 1**			**Sample 2**			**Sample 3**		
	**SEM**	**DNNAE**	**RE**	**SEM**	**DNNAE**	**RE**	**SEM**	**DNNAE**	**RE**
p	401	406	1.2	405	403	0.5	407	406	0.2
w_1	152	148	2.7	119	101	15.1	114	104	8.8
w_2	195	201	3.1	149	151	1.3	125	104	16.8
r	30	30	0	5	5	0	48	38	20.8
h	171	165	3.5	175	163	6.9	186	197	5.9
t_1	35	39	11.4	50	50	0	0	0	N/A
t_2	0	0	N/A	0	0	N/A	184	199	8.1

9. Implementation of compensation

Compensation was conducted in this study to enhance accuracy. The simplest linear form was chosen to ensure generalization capability. To determine the weight matrix and bias vector for linear compensation, uncompensated results were initially obtained by excluding the final step of the DNNAE method. Subsequently, the least squares (LS) method was applied to determine the optimized weight and bias factors for each grating parameter. The goal of the LS method was to minimize the MSEs between the observed values and true values. This process can be described as follows:

$$(w_i, b_i) = argmin \frac{1}{N_s} \sum_{j=1}^{N_s} \left\| w x_{i,j} + b - x_j^* \right\|_2 \tag{S3}$$

where N_s represents the number of samples, $x_{i,j}$ denotes the observed i^{th} grating parameter for the jth sample, and x^* corresponds to the true values. By solving Equation (S5) for each grating parameter, the weight matrix **W** = diag(w_1, w_2, …, w_9) and bias vector **b** = [b_1, b_2, …, b_9]^T were obtained. Notably, compensation was not applied to the starting azimuthal angle (φ^0) due to difficulties in determining the true values. It is important to mention that the weight matrix **W** and bias vector **b** are unique to different fabrication techniques, as certain errors are random and vary with fabrication methods and materials. For instance, the imprint resist (PBMA) experiences significant shrinkage under the bombardment of the electron beam, while silica exhibits minimal deformation under the same conditions. Six samples were fabricated using each fabrication method illustrated in Section 4, and four of them were employed to determine the compensation coefficients.

10. Characterization Results for Additional Samples

Figure S7 displays the characterization results for three additional samples. Sample 4 was patterned using NIL, sample 5 underwent etching using RIE, and sample 6 was patterned using LIL. The ellipsometric data generated by the DNNs, corresponding to the grating parameters inferred by the DNNAE method, closely aligns with the measured and simulated data. Additionally, the nano-grating profiles are accurately reconstructed, with MAEs of grating widths for the three samples being only 2.6 nm, 4.6 nm, and 3.8 nm, respectively.

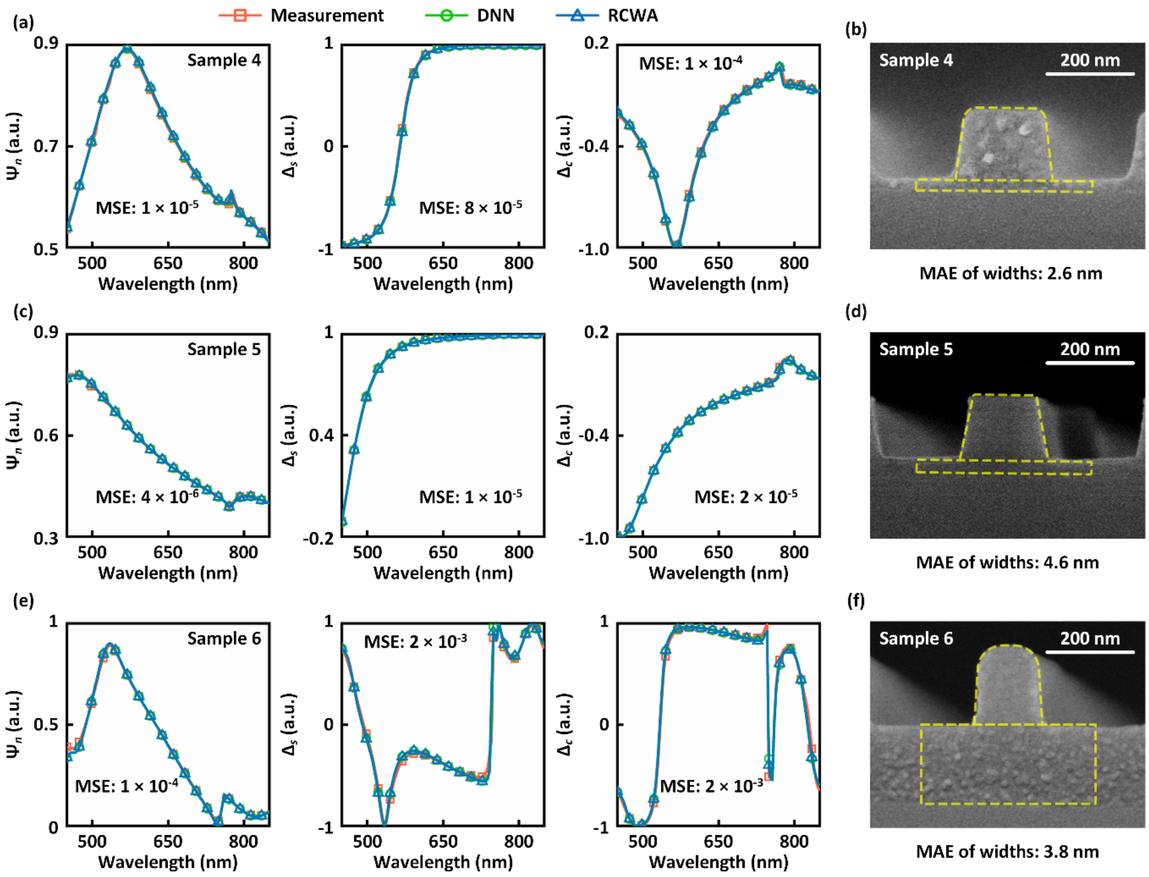

Figure S7. Characterization results for additional samples. (a), (c), (e) Comparison of ellipsometric data at the fifth measuring azimuthal angle for the nanoimprinted (sample 4), etched (sample 5), and lithographic (sample 6) nano-gratings, respectively. Measurement: ellipsometric data obtained from measurements. DNN: ellipsometric data generated by DNNs using the grating parameters inferred by the DNNAE method. RCWA: ellipsometric parameters simulated by RCWA using the grating parameters inferred by the DNNAE method. (b), (d), (f) Inferred profiles (yellow dashed lines) and SEM images for the nanoimprinted (sample 4), etched (sample 5), and lithographic (sample 6) nano-gratings, respectively.
